# Supplementary material for: HIVprotI: an integrated web based platform for prediction and design of HIV proteins inhibitors
Source: J Cheminform. 2018 Mar 9;10:12. doi: 10.1186/s13321-018-0266-y (PMC5845081; doi:10.1186/s13321-018-0266-y)
Supplement: Supplementary file 2 — Additional file 2. Source code of HIVProtI web server. [file 13321_2018_266_MOESM2_ESM.zip › HIVprotI_Source-code/hivprotI/weka-3-6-6/doc/weka/classifiers/trees/lmt/package-summary.html]

weka.classifiers.trees.lmt


---


|  |  |  |
| --- | --- | --- |
| **Overview** \| **Package** \| Class \| **Tree** \| **Deprecated** \| **Index** \| **Help** \| **Weka's home** | |  |
| **PREV PACKAGE**   **NEXT PACKAGE** | **FRAMES**    **NO FRAMES**     **All Classes** |  |


---

## Package weka.classifiers.trees.lmt

| **Class Summary** | |
| --- | --- |
| **LMTNode** | Class for logistic model tree structure. |
| **LogisticBase** | Base/helper class for building logistic regression models with the LogitBoost algorithm. |
| **ResidualModelSelection** | Helper class for logistic model trees (weka.classifiers.trees.lmt.LMT) to implement the splitting criterion based on residuals. |
| **ResidualSplit** | Helper class for logistic model trees (weka.classifiers.trees.lmt.LMT) to implement the splitting criterion based on residuals of the LogitBoost algorithm. |

---


|  |  |  |
| --- | --- | --- |
| **Overview** \| **Package** \| Class \| **Tree** \| **Deprecated** \| **Index** \| **Help** \| **Weka's home** | |  |
| **PREV PACKAGE**   **NEXT PACKAGE** | **FRAMES**    **NO FRAMES**     **All Classes** |  |


---
